# Distinct mental trainings differentially affect altruistically motivated, norm motivated, and self-reported prosocial behaviour

**DOI:** 10.1038/s41598-018-31813-8

**Published:** 2018-09-10

**Authors:** Anne Böckler, Anita Tusche, Peter Schmidt, Tania Singer

**Affiliations:** 1 Max Planck Institute for Human Cognitive and Brain Sciences, Leipzig, Germany; 2 Julius-Maximilians-Universität Würzburg, Würzburg, Germany; 3 Division of the Humanities and Social Sciences, California Institute of Technology, Pasadena, CA USA; 4 Justus-Liebig-Universität Gießen, Gießen, Germany; 5 Cardinal Wysczinski University, Warsaw, Poland

## Abstract

Global challenges such as climate change or the refugee crises emphasize the necessity of altruism and cooperation. In a large-scale 9-month intervention study, we investigated the malleability of prosociality by three distinct mental trainings cultivating attention, socio-affective, or socio-cognitive skills. We assessed numerous established measures of prosociality that capture three core facets: Altruistically motivated behaviours, norm motivated behaviours, and self-reported prosociality. Results of multiple time point confirmatory factor analyses support the validity and temporal stability of this model. Furthermore, linear mixed effects models reveal differential effects of mental trainings on the subcomponents of prosociality: Only training care and compassion effectively boosted altruistically motivated behaviour. No effects were revealed for norm-based behaviour. Self-reported prosociality increased with *all* training modules; this increase was, however, unrelated to changes in task-based measures of altruistic behaviour. These findings corroborate our motivation-based framework of prosociality, challenge economic views of fixed preferences by showing that socio-affective training boosts altruism, and inform policy makers and society about how to increase global cooperation.

## Introduction

Human prosociality is at the heart of peaceful societies and key to facing global challenges such as the fair distribution of finite resources, slowing down climate change, or helping millions of refugees to a life in safety and dignity. Research on cooperation and altruism has been the focus of many disciplines ranging from philosophy and psychology^1,2^, to mathematics and economy^3–8^, evolutionary biology^6,9–11^, and neuroscience^12–17^. Yet, surprisingly little is known about whether and how human altruism can be ‘trained’, likely because economic models often consider prosociality as stable social preference^18–20^ which seems to be, in part, genetically determined^21,22^. However, emphasizing the role of nurture besides nature, longitudinal evidence demonstrates that experimentally induced changes of the social environmental can increase prosociality in children^23^.

With growing interest in effects of secularized contemplative mental training programs such as Mindfulness-Based Stress Reduction (MBSR^24^), or the Mindful Self-Compassion Program (MSC^25^), recent studies have also started to address the influence of mindfulness-, compassion-, or loving-kindness-based trainings on adults’ prosocial behaviours, such as charitable donations, helping, or contributions in an economic decision-making task^26–30^. These initial findings suggest that specific prosocial behaviours might indeed be malleable through contemplative mental training.

Crucially, both mental training and human prosociality are complex and heterogeneous constructs. Contemplative mental interventions typically involve numerous different practices, and prosocial behaviours can be motivated and expressed in various ways, limiting conclusions based on mixed training programs such as MBSR^24,25^ or based on single markers of altruism^1,5,6,9–11,15,28,31^. To systematically address *if*, *how*, and to *what degree* prosocial behaviour is enhanced by mental training, we need to investigate the *specific* effects of different types of trainings on *distinct* facets of human prosociality. In the following, we introduce a framework of prosociality that does justice to the heterogeneity of the phenomenon, and a conceptual framework of mental training that differentiates specific types of mental practices.

**Figure 1:**
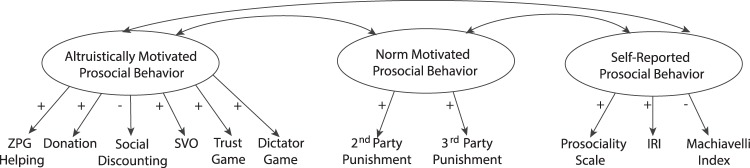
Structure of human prosociality. The figure schematically illustrates the proposed relationship of various prosocial measures and three latent variables of prosociality^34,35^. ZPG: Zurich Prosocial Game, SVO: Social Value Orientation, IRI: Interpersonal Reactivity Index. +/− indicate positive/negative standardized regression weights. Note that the variables 2^nd^ and 3^rd^ Party Punishment were constrained to equality. Note that the variable Social Discounting was modelled to also load on the factor Self-Reported Prosocial Behaviour in^35^. This relation was added in a data-driven manner (indicated by model modification indices) and is not depicted here, nor was it modelled in the analyses of the present study.

Prosocial behaviour is defined as behaviour that is costly to the individual and benefits others at the individual or group-level^32^. Directly speaking to recent debates whether similar or different *motivations* underlie several types of prosocial *behaviours* (e.g.^33^), we recently assessed various measures of prosocial behaviour from different research disciplines ranging from psychology to micro-economy and subjected them to factor analyses^34,35^. This data-driven approach structured the measures of prosociality according to three distinct latent factors (Fig. 1): *Altruistically motivated* prosocial behaviour that aims to enhance others’ well-being even at a cost to oneself; *norm motivated* prosocial behaviour representing the tendency to enforce social norms through costly punishment; and *self-reported* prosocial behaviour as the disposition or motivation to perceive and/or describe oneself as moral, generous, and helpful. All three factors are theoretically rooted^6,9–11,15,18,31^, empirically sound, and show clear-cut and differential relations to affective and cognitive dispositions^34,35^. The factor altruistically motivated prosocial behaviour encompassed various measurement methods, including game theoretical paradigms like the Dictator Game and the Trust Game^5,36^, real-world charitable donations^37^, psychological assessments of spontaneous helping^28^, and self-reported distribution choices indicating social value orientation and social discounting^38,39^, suggesting that the factors capture motivation-based constructs rather than mere method variance^40^. Hence, this framework of prosociality allows to test whether specific types of mental interventions differentially affect the motivations underlying prosocial behaviour.

Mindfulness- and compassion-based contemplative practices are of particular interest as a tool for mental interventions boosting human prosociality as these have been shown to alter prosocial behaviours^26–29^ and have beneficial effects on health, stress, well-being, and brain structure (e.g.^41–43^). Because mindfulness- and compassion-based programs such as MBSR or MSC^24,25^ typically include various types of mental practices, we recently proposed a classification of these practices according to the underlying psychological mechanisms (see^44–46^). In short, our model distinguishes three broad classes of mental capacities targeted by mental trainings: 1) present-moment attention and body-awareness, 2) socio-affective skills such as compassion, gratitude, and prosocial motivation, and 3) socio-cognitive capacities such as meta-cognition and perspective taking on self and others.

**Figure 2:**
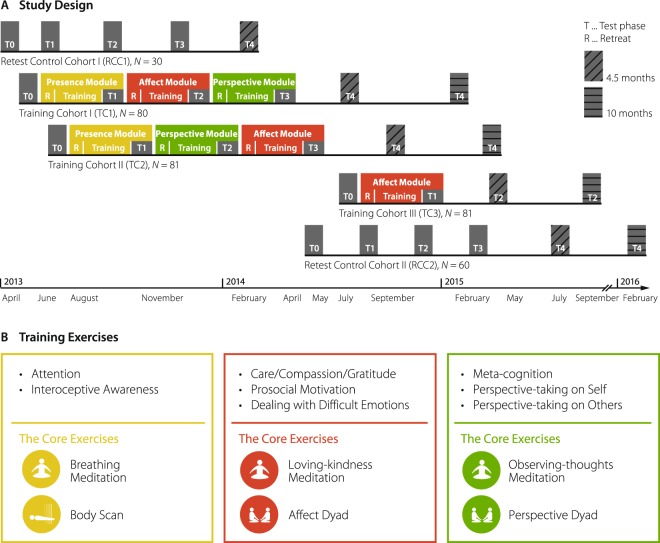
Design and training exercises of the *ReSource* study. Panel (A) Design and timeline of the longitudinal study. Training and testing took place from April 2013 until February 2016. The timelines for retest control cohorts (RCC1 and RCC2) and training cohorts (TC1, TC2, TC3) are represented one below the other. Training modules in the training cohorts are depicted by coloured areas (yellow for the Presence Module; red for the Affect Module, and green for the Perspective Module); data collection phases are depicted by grey areas (T0-T4). Specifically, TC1 and TC2 completed all three training modules and differed only in the order of the Affect and Perspective Module. TC3 only completed the Affect Module. R in coloured boxes indicates the retreats that took place in the beginning of each module. RCC1 and RCC2 were split for logistical reasons into two smaller cohorts but were jointly analysed. Both retest control cohorts completed all measurements but did not receive any training. Panel (B) Illustration of the trained skills and the core exercises of the three modules (left to right): Presence (yellow), Affect (red), Perspective (green). Source: Figure courtesy of^46^.

The present study was realized in the context of the *ReSource Project*, a large-scale 9-month longitudinal mental training study^46^ and incorporated both, the multi-faceted concept of prosociality as well as the refined framework of mental training. This allowed investigating how distinct mental trainings affect different facets of prosocial behaviour. Specifically, the *ReSource Project* implemented three mental training modules: *Presence* focusing on the cultivation of present-moment attention and interoceptive awareness (practices included in most mindfulness-based intervention programs^24^); *Affect* focusing on the enhancement of socio-affective skills such as care, compassion, gratitude, and prosocial motivation (included in loving-kindness based interventions^47^); and *Perspective* aiming at increasing socio-cognitive skills such as perspective taking on self and others as well as meta-cognitive awareness (for details about scientific and contemplative background see^46^). Figure 2 depicts the training schedule and respective exercises. To test for the impact of these distinct training modules on the three sub-components of prosociality, we implemented them together with a comprehensive battery of thirteen prosocial measures (see Table 1) in a large and representative participant sample (N = 332)^46^. Assessments of prosocial behaviour were completed at baseline (T0, before any form of mental training) and after each training module at three, six, and nine months (T1, T2, T3; see Fig. 2).

**Table 1:** Short description of included measures of prosociality.

	Description	Measure
***Game Theoretical Paradigms***	*Computerized tasks were performed using an online gaming platform that connected participants to a pool of anonymous players. Participants played for monetary units (MUs, increments of 1) that were later transferred into real money (1MU = 10 Eurocents). All economic games were implemented as one-shot versions*.	
Dictator Game (DG)^5^	Participants were informed about MUs at their disposal and choose how many MUs they wanted to give to Player B (2 rounds).	Mean % of given MUs
Trust Game (TG)^36^	Participants were informed about MUs and chose how many MUs to invest in the other player (TG, 1 round)	% invested MUs in TG
2^nd^ Party Punishment Game (2^nd^ PPG)^67^	Participants were informed about MUs and then Player A chose how many MUs to transfer to Player B; Player B could then invest MUs to punish Player A (1 invested MU = −3 MU Player A). Participants played 2 rounds as Player A, followed by 3 rounds as Player B.	(a) Mean % MUs invested as Player B (=2^nd^ person punishment) (b) Mean % of MUs given as Player A in 2^nd^ PPG minus mean % of MUs given in DG (=strategic giving)^15^
3^rd^ Party Punishment Game (3^rd^ PPG)^67^	Participants were informed about Player A’s endowment, saw how many MUs Player A transferred to another anonymous Player B, and had the possibility to punish Player A (1 invested MU = −3 MU Player A) (3 rounds).	Mean % of invested MUs to punish Player A
***Interactive Computer Tasks***	*Participants completed these tasks via an online platform playing for real money*. *Decisions of participants to help or benefit others directly affected their own payoffs*.	
Zurich Prosocial Game (ZPG)^28^	Participants navigated a figure through a maze to receive a treasure (=50 Eurocents), using a limited number of keys to remove obstacles from their path (or from another player who moved on a separate route).	(a) Overall helping (% of keys invested to remove obstacles from the other player’s paths) (b) Cost effect (% of times helped when helping was costly minus not costly because participants couldn’t use keys for themselves anymore)
Donation Task^37^	Participants saw short descriptions of charities and indicated how much of their endowment (50 €) they wanted to donate (8 trials, 1 randomly chosen and implemented at the end of the task).	Mean % donations
***Hypothetical Distribution Tasks***	*These scales were filled in online to minimize demand effects and were based on hypothetical distribution choices only*.	
Social Discounting^38^	For each of 7 imagined acquaintances of different social distances to them, participants made 9 distribution choices (either selfish of generous; crossover point between last selfish and first altruistic choice represents the amount they were willing to forgo for an acquaintance).	Degree of discounting (k) (assuming hyperbolic function between social distance and amounts participants were willing to forgo)
Social Value Orientation Scale (SVO)^39^	Participants choose between three distribution options (prosocial = optimizing other’s gain; individualistic = optimizing one’s own gain; competitive = maximizing the difference in gains) (9 rounds).	Number of prosocial choices
***Psychological Trait Questionnaires***	*Trait questionnaires were filled in via an internet platform*, *minimizing demand effects*.	
Prosocialness Scale^80^	Assesses propensity to help and support others, e.g., “I am available for volunteer activities to help those who are in need”.	Subject-specific mean scores
Machiavelli Scale^81^	Assesses tendency to favor strategic self-interest over moral-based behaviour, e.g., “Acquaintances should be selected according to whether they are beneficial”.	Subject-specific sum scores
Interpersonal Reactivity Index (IRI)^82^	Assesses empathic concern, personal distress, perspective taking, and empathic fantasy, e.g., “When I see someone being taken advantage of, I feel protective towards them”.	Subject-specific sum scores

This setup enables us to validate our proposed framework of human prosociality, access its stability over time, and, most importantly, identify the distinct types of mental practices that can effectively enhance the different motivation-based facets of prosociality. Identifying the types of mental practice that enhance altruism has wide ranging implications for human society. Policy makers and the public alike struggle with the question of how global cooperation can be improved effectively and reliably to deal with global challenges such as climate change or depletion of natural resources. Especially mental trainings that effectively increase altruistic motivation may offer an innovative approach to complement current political attempts to encourage acts of prosociality that range from punishing violations of fairness norms (e.g., defraudation of tax) to actively incentivising generosity (e.g., the possibility to set off donations against tax liability).

## Results

Descriptive results of all prosocial measures obtained from T0 to T3 for each experimental cohort are depicted in Supplement S1.

### Validation and measurement invariance of the proposed structure of human prosociality

We subjected the measures of prosociality to multiple time points confirmatory factor analyses (MT-CFA) according to our proposed three-factor structure (Fig. 1, for details, see method section). This method allows 1) validating the proposed factor structure, 2) probing temporal stability of the model and the latent factors of prosociality, and 3) investigating longitudinal measurement invariance of the model, which is a precondition for investigating training induced change in the scores of the latent factors of prosociality^48^. Table 2 presents the fit indices for all measurement models. Regression weights and correlation coefficients are depicted in Supplementary Material S2. Results of the MT-CFA (configural invariance model) showed adequate fit to the data (CFI = 0.93; TLI = 0.91; RMSEA = 0.04), supporting the validity of our proposed structure of human prosociality^34,35^ and demonstrating its temporal stability. The metric invariance model restricting all factor loadings to be equal across time, in addition to constraints specified in the configural model, showed an adequate fit to the data (CFI = 0.93; TLI = 0.91 RMSEA = 0.04) with no evidence of deterioration of fit (reduction of CFI < 0.01), suggesting metric measurement invariance^48,49^. The scalar invariance model additionally restricting all intercepts to be equal across time also resulted in an adequate model fit (CFI = 0.92; TLI = 0.91; RMSEA = 0.04) and no deterioration of model fit (reduction of CFI < 0.01), suggesting scalar measurement invariance^48,49^. Measurement invariance implies that the same latent constructs are measured in the same way across time. This allows for meaningful interpretation of change in means (scalar invariance) and covariances of latent factors (metric invariance) and, hence, is a necessary prerequisite for addressing differential and training induced change^48^. Measurement invariance was also demonstrated when additional parameters were freed by specifying autocorrelated covariances of all items to be equal across time^50^ to account for limitations due to sample size (n = 332) (see Table 2). All latent factors of prosociality showed significant autocorrelations across all time points (rs ≥ 0.53, ps ≤ 0.002). No significant correlations between the factors of prosociality were revealed at any time point (rs ≤ 0.07, ps ≥ 0.066).

**Table 2:** Fit indication for the multiple time point confirmatory factor analyses.

	χ^2^	df	RMSEA	TLI	CFI	∆ CFI
**Original model (three latent factors of prosocial behaviour)**						
Configural invariance	1269.44	810	0.041	0.914	0.930	—
Metric invariance	1300.23	831	0.041	0.914	0.928	−0.002
Scalar invariance	1384.97	854	0.043	0.906	0.919	−0.009
**Model with autocorrelated covariances of items constrained to be equal over time**						
Configural invariance	1396.51	865	0.043	0.907	0.919	—
Metric invariance	1425.64	886	0.043	0.908	0.917	−0.002
Scalar invariance	1528.50	910	0.045	0.897	0.905	−0.012

χ^2^ = values of the Likelihood Ratio Test (chi-square); df = degrees of freedom**;** RMSEA = Root Mean Square Error of Approximation; TLI = Tucker-Lewis-Index; CFI = Comparative Fit Index.

### Re-test reliability of factor scores in retest controls (RCC)

To provide further evidence for the temporal stability of estimated factor scores, we assessed the re-test reliability of prosocial factor scores in the cohort that did not undergo any mental training (retest control cohort, RCC). Note that a longitudinal CFA based approach to assessing retest reliability was precluded by the relatively small number of control participants (n = 90). Altruistically motivated and self-reported prosocial behaviour showed high re-test reliability across all timepoints (mean re-test reliabilities were 0.69 and 0.86 respectively). Norm motivated prosocial behaviour displayed medium sized to large re-test values (mean re-test reliabilities of 0.52). See Table 3 for details.

**Table 3:** Re-test reliability (correlation coefficients) for scores on the three factors of prosociality in the retest control cohort (RCC).

	T1	T2	T3
**Altruistically Motivated Prosocial Behaviour**			
T0	0.667^***^	0.567^***^	0.477^***^
T1		0.685^***^	0.501^***^
T2			0.751^***^
**Norm Motivated Prosocial Behaviour**			
T0	0.217^*^	0.251^*^	0.266^*^
T1		0.515^***^	0.404^***^
T2			0.631^***^
**Self-Reported Prosocial Behaviour**			
T0	0.851^***^	0.845^***^	0.802^***^
T1		0.896^***^	0.879^***^
T2			0.877^***^

*** Indicates significant correlations at p < 0.001 (2-tailed), *p < 0.05 (2-tailed).

### Training-induced changes in prosociality

Having established the robustness and measurement invariance of the factor structure of human prosociality over time, we examined training-induced changes in scores of each of the prosocial factors. In short, based on results of the longitudinal CFA, the eleven individual measures of prosociality were integrated according to our factor model so that we received one score for each factor of prosociality at each time point (T0–T3) (for details, see method section). Linear mixed-effects models (LMM) were specified for each sub-component of prosociality for the between-subject factor *group*, the within-subject factor *time*, and random intercepts for participants. LMMs are the most straightforward approach to address our question of specific mental training effects on the sub-components of prosociality and they are especially suitable for our study, because they are robust to unbalanced designs and are particularly proficient in giving unbiased results in the presence of missing data/attrition^51^. Within each LMM, we tested whether factor scores across groups were comparable at T0 and whether the groups changed differentially over time (interaction *group* and *time*). Only if both were the case, we further assessed which training module (Presence, Affect, Perspective) was effective in inducing change by comparing the average increase in factor scores due to each module against zero (one sample t-test, two-tailed) and against the average change in factor scores in the RCC from T0 to T3 (independent sample t-test, two-tailed) (see method section for details). For each group of t-tests, Benjamini-Hochberg corrections^52^ were applied to correct for multiple testing. The result section reports test statistics of the LMMs (F values, degrees of freedom), test statistics of t-tests (t values, degrees of freedom), and p values (the probability of obtaining a result equal to or more extreme than the observed result, given that the null-hypothesis is true). Cohen’s d provides a measure of effect size.

#### Altruistically motivated prosocial behaviour

**Figure 3:**
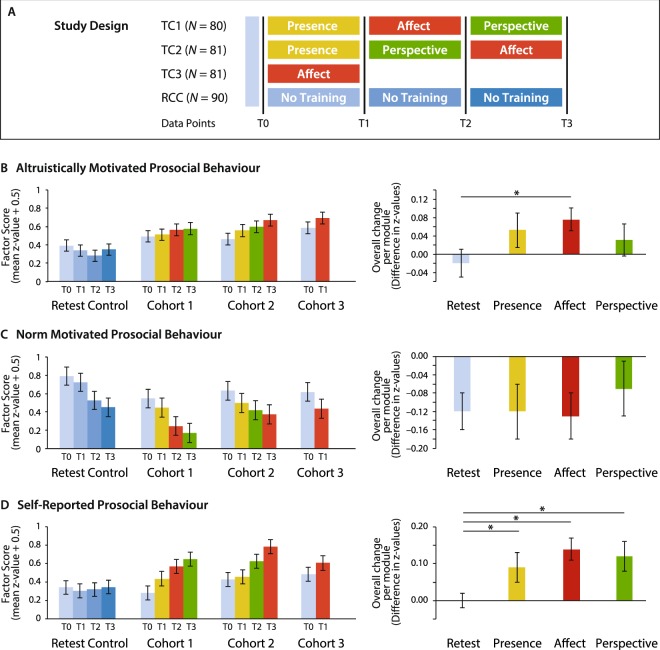
Results. Panel (A) Schematic depiction of the study design. Four cohorts were tested, three training cohorts (TC1, TC2, and TC3) and a retest control cohort (RCC). Colour coding in all panels is in accordance to this scheme, showing results for the RCC in blue, results after the Presence Module in yellow, results after the Affect Module in red, and results after the Perspective Module in green. Panel (B) Left graph: Results for scores on altruistically motivated prosocial behaviour for all cohorts and all time points. The y-axis displays factor scores as mean z-value + 0.5 (preventing negative scores and enhancing readability). Standard errors are displayed. The cohorts did not significantly differ in factor scores at T0. While no significant increases in factor scores were revealed for the RCC and Cohort 1 (TC1), TC2 and TC3 showed significant increases of altruistically motivated behaviour over time. Right graph: Average change scores of the factor altruistically motivated prosocial behaviour for the RCC and the three training modules of the ReSource Study. * depict significant differences from RCC (p < 0.05), corrected for multiple comparisons. Standard errors are displayed. Panel (C) Results for scores on norm motivated prosocial behaviour. Cohorts did not significantly differ in factor scores at T0. All cohorts showed a significant decrease in factor scores over time. Panel (D) Results for scores on the factor self-reported prosocial behaviour. Cohorts did not significantly differ in factor scores at T0. The RCC did not show significant change in factor scores over time, while all training cohorts did.

Factor scores of each cohort and each time point are depicted in Fig. 3B (left graph). Overall effects per training module are depicted in Fig. 3B (right graph). No significant effect of *group* was revealed at T0 (F(3, 529.71) = 1.7, p = 0.17), suggesting that levels of altruistic behaviour were comparable at baseline, which is crucial to assess differential effects of distinct training modules. Overall, we found a significant main effect of *time* (F(3, 535.24) = 2.79, p = 0.040), reflecting an increase of factor scores over the course of the training. The main effect of *group* was also significant (F(3, 338.02) = 5.96, p = 0.001), pointing towards differences in altruistically motivated behaviour between cohorts. The significant two-way interaction of *time* and *group* (F(7, 597.52) = 2.09, p = 0.042) suggests different effects of time across cohorts: While the RCC showed no increase in altruistic behaviour over time (F(3, 475.35) = 1.4, p = 0.22), the majority of training cohorts did show increasing altruistic behaviour over time (TC1: F(3, 475.53) = 0.72, p = 0.53; TC2: F(3, 473.73) = 3.8, p = 0.011; TC3: F(1, 717.55) = 4.6, p = 0.032).

Module-specific effects on scores of altruistically motivated prosocial behaviour were assessed by comparing overall effects for each module against zero (one sample t-test) and against the average increase in the RCC (independent sample t-test). See Fig. 3B (right graph). Testing module effects against zero revealed a significant increase in altruistic behaviour due to the Affect Module (t(229) = 3.00, p = 0.003, d = 0.20; surviving correction for multiple comparisons). No other effects reached significance (ps ≥ 0.16). Similarly, compared to change scores in the RCC, altruistically motivated prosocial behaviour was significantly increased after Affect (t(199.31) = 2.51, p = 0.013, d = 0.30; surviving correction for multiple comparisons). Because the Levene’s test for equality of variances revealed violated assumptions of homogeneity (F(1, 313) = 16.13, p = 0.000), the present analysis is based on adjusted t statistics not assuming equality of variances. No other training module had a significant effect on altruistic behaviour when compared to RCC (ps ≥ 0.11). These results suggest that three months of cultivating gratitude, care, and compassion significantly enhanced participants’ altruistically motivated prosocial behaviour (showing small (d = 0.20) or small-to-medium (d = 0.30) effect sizes^53^). By contrast, practicing interoceptive awareness and present-moment attention (Presence) or practicing perspective taking and metacognition (Perspective) did not systematically influence this component of prosocial behaviour.

In order to probe whether the influence of the Affect Module on altruistically motivated prosocial behaviour was further modulated by the preceding training, that is, whether Affect is specifically influential after Presence (TC1), after Perspective (TC2), or with no preceding other training (TC3), we directly compared the specific change induced by the Affect Module between the three training cohorts by means of independent sample t-tests. No differences were revealed (ps ≥ 0.46), suggesting that Affect-induced changes in altruistically motivated prosocial behaviour were not systematically affected by the type of preceding training.

#### Norm motivated prosocial behaviour

Factor scores of each cohort and time point are depicted in Fig. 3C (left graph). Overall effects per training module are depicted in Fig. 3C (right graph). No significant differences in norm based behaviour were revealed at baseline (F(3, 579.36) = 1.06, p = 0.367). Results of the LMM showed a main effect of *time* (F(3, 515.49) = 11.89, p = 0.000), reflecting a general decrease in norm-based prosocial behaviour over time. Neither the main effect of *group* nor the two-way interaction of *time* and *group* reached significance (Fs < 1.96, ps ≥ 0.12). Taken together, these results suggest that norm motivated prosocial behaviour generally deteriorated over time, but was not systematically affected by the meditation-based trainings or by their absence (as implemented in the retest control cohort).

#### Self-reported prosocial behaviour

Factor scores are depicted in Fig. 3D (left graph) for each cohort and time point. Overall training effects per module are depicted in Fig. 3D (right graph). When the four cohorts were compared at T0, no significant effect of *group* was revealed (F(3, 447.21) = 1.40, p = 0.242), suggesting that there were no differences in self-reported prosocial behaviour across cohorts at baseline. Results of the LMM showed significant main effects of *time* (F(3, 528.90) = 20.84, p = 0.000) and *group* (F(3, 325.22) = 3.73, p = 0.012), suggesting an increase of self-reported prosociality over time as well as differences across cohorts. The two-way interaction of *time* and *group* was also significant (F(7, 554.97) = 4.87, p = 0.000). This interaction was due to increasing factor scores over time for all training cohorts (TC1: F(3, 453.61) = 14.55, p = 0.000; TC2: F(3, 440.36) = 14.75, p = 0.000; TC3: F(1, 634.91) = 5.61, p = 0.018), but not in the RCC (F < 1), suggesting that changes in self-reported prosocial behaviour were due to training effects rather than the repeated implementation of the respective measures.

Results of module-specific simple t-tests revealed that self-reported prosocial behaviour increased significantly for all three training modules (Presence: t(149) = 2.55, p = 0.012, d = 0.21; Affect: t(220) = 4.55, p = 0.000, d = 0.31; Perspective: t(147) = 3.43, p = 0.001, d = 0.28; all surviving correction for multiple comparisons), but not for the RCC (t < 1). Significant effects of training were confirmed when contrasting module-specific increases in self-reported prosociality against change-scores obtained for the RCC. Because the Levene’s test revealed violations of the assumption of homogeneity (Presence and RCC: F(1, 232) = 52.11, p = 0.000; Affect and RCC: F(1, 303) = 35.09, p = 0.000; Perspective and RCC: F(1, 230) = 39.22, p = 0.000; all surviving correction for multiple comparisons), all analyses are based on adjusted t-tests not assuming equality of variances: Presence: t(218.20) = 2.20, p = 0.029, d = 0.27; Affect: t(302.21) = 3.79, p = 0.000, d = 0.40; Perspective: t(218.29) = 2.94, p = 0.004, d = 0.36. Taken together, these findings indicate that meditation-based practices led participants to describe themselves as increasingly prosocial (reflected in small to medium effect sizes for each module^53^), independent of the target of the mental training practice. Further examination of this continuous increase of self-reported prosociality showed that effect sizes (compared against baseline, T0) changed from an average of d = 0.23 (small) for the first three months (T0 to T1) to d = 0.53 and d = 0.64 (medium-to-large) for six and nine months (T0 to T2; T0 to T3).

Next, we examined whether the effects of Affect and Perspective depended on the type of preceding training module. To this end we compared the effects of Affect and of Perspective on self-reported prosocial behaviour between the training cohorts. No significant differences were revealed (ps ≥ 0.20, independent-sample t-tests), suggesting no effects of training order.

#### Measures of strategic prosocial behaviour

Note that the original model of human prosociality also included measures assessing *strategic* prosocial behaviour, related to the inclination to make prosocial choices dependent upon whether they benefit oneself^34^. However, further investigation is required to confirm whether a separate strategy factor can be established^35^. For the sake of completeness and future studies on strategically motivated prosocial behaviour, we also provide details on individual measures of strategic prosocial behaviour included in the ReSource Project and their training-induced plasticity (Supplement S4).

Because the current study aimed to test which mental training modules effectively enhance each of the three facets of prosociality, we compared effects of the three mental training modules to RCC. Results of comparisons between the training modules are reported in Supplement S5.

### Correlations between training-induced changes in different facets of prosociality

In addition to testing differential effects of the three training modules on the identified factors of human prosociality, we tested for systematic links between training-induced changes across the prosocial factors by subjecting them to correlation analyses. We estimated the average training-induced effects across the entire span of training for each participant and each facet of prosociality, independent of the module (i.e., for TC1 and TC2: mean of T1-T0, T2-T1, and T3-T2; for TC3: T1-T0). This approach was chosen for the sake of comparability between cohorts with nine months of training (TC1 and TC2) and the cohort with only three months of training (TC3). We report Pearson correlation coefficients (r) and p values. Benjamini-Hochberg corrections^52^ were applied to correct for multiple testing.

Training-induced changes on altruistically motivated behaviour were not correlated with training-induced changes in self-reported prosocial behaviour (r = −0.02, p = 0.76). Hence, people who perceived themselves as increasingly prosocial (assessed through psychological trait questionnaires) were not necessarily the same people who behaved more altruistic in task-based assessments of prosocial behaviour. Interestingly, however, individual differences in training-induced increases in altruistically motivated behaviour were related to individual differences in norm-enforcing behaviour (r = 0.25, p = 0.000, surviving correction for multiple comparisons). This finding suggests that the more individuals increased in altruistic behaviour due to the training, the less they decreased in enforcing social norms at a cost to themselves. No other significant correlations were revealed between training-induced changes across the three factors of prosociality (all ps > 0.1). No significant relations between the factors of prosociality were revealed in the RCC (ps ≥ 0.043, not surviving correction for multiple comparisons).

### Summary

First, results of longitudinal CFAs revealed the validity, temporal stability, and measurement invariance of our proposed three-factor structure of human prosociality. Second, all three factors of prosociality showed good retest-reliability. Third, LMMs and subsequent posthoc tests demonstrated that only the Affect Module effectively enhanced altruistically motivated prosocial behaviour when compared to the retest control group. This Affect-induced increase in altruistic behaviour showed a small effect size and was independent of training order. None of the training modules affected norm motivated prosocial behaviour beyond the decline in punishment behaviours observed in the retest control group. All training modules effectively enhanced self-reported prosocial behaviour with no evidence in favour of order effects. Effect sizes of these module-specific increases were small-to-medium, but when increases in self-reported prosociality were probed over time, effect sizes changed from small after three months of training to medium after six and nine months of training. Finally, training-induced increases in altruistically motivated prosocial behaviour were uncorrelated to training-induced changes in self-reports of prosociality, but negatively correlated with decreases in norm motivated prosocial behaviour.

## Discussion

The present findings validate our proposed framework of human prosociality and demonstrate temporal stability of all three latent factors: Altruistically motivated, norm motivated, and self-reported prosocial behaviour^34,35^. Furthermore, the results provide compelling evidence that human prosociality is malleable and that distinct facets of prosociality can be systematically shaped by different types of mental trainings, consisting of short daily practices that can be easily implemented in everyday life. Specifically, we show that only the compassion- and care-based mental training boosted altruistically motivated behaviours. By contrast, subjective self-reports of prosociality, as assessed with psychological trait questionnaires, increased irrespective of training type, but this increase was not related to changes in decision-based altruism. Norm-based behaviours were not affected by any of the mental trainings.

Based on previous findings revealing that changes in the social environment and meditation-based interventions can enhance prosocial behaviours^23,26–29^, a question of particular interest was which mental training would prove effective in enhancing altruistically motivated behaviour. We found that altruistically motivated prosocial behaviour was selectively augmented by cultivating an affective-motivational route of social understanding^54^ that targeted qualities such as care, gratitude, and compassion as well as accepting difficult emotions (Affect Module). This result extends initial evidence of effects of mindfulness-, compassion-, and loving-kindness interventions on single measures of prosociality (e.g.^26–30^) and of findings demonstrating the impact of changing the social environment on prosociality in children^23^. Specifically, we show that the socio-affective training impacts the latent factor underlying various altruistic behaviours in adults, ranging from generosity and trust as measured through game theoretical paradigms^5,36^, spontaneous helping as assessed in an implicit computer-based task^28^, donations for real-world charitable organizations, self-report-based social value orientation^39^, and peoples’ tendency to disregard social closeness during prosocial decision-making (i.e., low social discounting^38^). This finding suggests that the Affect Module directly affects the *motivation* underlying this type of prosocial behaviours. In fact, the mental practices of the Affect Module might successfully activate a psycho-biological system associated with care and affiliation and linked to guaranteeing survival of offspring through parental bonding^55^. Animal and human research has linked the neuropeptide oxytocin to these functions, indicating a role of oxytocin in bonding, prosocial behaviours, empathy, and social cognition^56,57^. Consistently, evidence from our lab and from within the *ReSource Project* revealed that expertise in compassion meditation and compassion training in novices are linked to enhanced positive affect, increased brain activity in regions associated with reward and affiliation that contain oxytocin receptors, and structural plasticity in brain areas involved in socio-affective processing^41,58,59^.

Our data further indicate that training effects did not differ depending on whether the Affect Module was implemented initially, after three month, or after six months of training. Hence, the Affect Module, consisting of three introductory days, weekly meeting with a teacher, and about 30 minutes of daily practice over the course of three months, effectively boosted altruistic behaviours independent of how those exercises were combined with other practices. In addition, our results have important implications for the growing field of contemplative mental training research^44,60–62^ as they provide a bench mark for effect sizes of training effects. We show, for instance, that the effect of the socio-affective training on altruistic behaviour can be classified as small^53^, a fact that can easily lead to over-estimations when sample sizes are small^63^. A critical question for future research will be to investigate to what degree the duration of trainings matter. For instance, would six or nine months of socio-affective training be reflected in medium or even large effect sizes? In addition, future studies should address the replicability of present training-effects and extend them to settings where control groups receive non-contemplative interventions.

Interestingly, the Perspective Module that targeted a ‘cold’ cognitive route to understanding others failed to enhance altruistically motivated behaviour (for a distinction of socio-cognitive and socio-affective routes in brain and behaviour, see^54,64^). This finding is somewhat surprising in light of traditional neoclassical economic views advocating that cognitive understanding and rational thinking is the most promising route to increased cooperation^65^. However, our results are consistent with recent findings showing that socio-affective processes like empathic concern are a stronger predictor for altruistic behaviour such as charitable donations than cognitive perspective taking^37^. Our data are also relevant for the ongoing debate on whether mindfulness practices that focus on present-moment awareness and attention (as targeted in the Presence Module) are sufficient to elicit compassion and altruism, or if such other-related ethical qualities can only be enhanced by mental practices that specifically target ‘qualities of the heart’ and care- and affiliation-based systems^59,66^. We found no significant increases in altruistic behaviour due to the Presence Module, indicating that practices focusing only on present-moment attention are not efficient in altering altruistic motivations, at least not with three months of practice. It is important to note that many MBSR teachers instinctively combine the attention-based practices that were implemented in the Presence Module with components of self-compassion and kindness. Future studies should therefore continue to tease apart effects of the various meditation-based practices on altruism.

In our study, norm motivated prosocial behaviour generally deteriorated over time both in the training groups as well as the re-test control group, suggesting that familiarization with the respective tasks led people to decrease monetary investments to enforce social norms. This is consistent with evidence from behavioural economics also showing generally decreasing monetary investments over time^4^. Importantly, neither the cultivation of attention-based (Presence), nor of socio-affective (Affect), or socio-cognitive capacities (Perspective) systematically shaped this behaviour. However, when focusing on individual differences, training-induced increases in altruistically motivated behaviours were positively related to slower decreases in norm-motivated behaviour. This finding points towards a positive link between enhancing others’ well-being and strengthening social norms at a cost to oneself. Hence, even though altruistic and norm-based behaviours reflect different underlying motivations, shifts towards the motivation to care for and support others may be linked to shifts towards the motivation to help establish social norms^67^.

Finally, participants described themselves as increasingly prosocial as training progressed, irrespective of training modules. Thus, contrary to specific effects of the Affect Module on altruistically motivated prosocial behaviours, self-reported prosociality assessed through trait-questionnaires was also enhanced by mental practices that focused on present-moment attention or socio-cognitive skills. Importantly, training-induced changes in self-reported prosocial behaviour did *not* correlate with behaviour-based increases in altruistically motivated behaviour. This finding is consistent with cross-sectional evidence suggesting that self-reports as assessed in trait-questionnaires represent a distinct component of human prosociality^34,35^. These findings have practical implications given that trait-questionnaires and behaviour-based measures are often used interchangeably. However, we cannot rule out that the continuous increase in self-reported prosociality irrespective of training module may, at least partly, reflect social desirability effects previously discussed in the context of subjective self-report measures^68^.

Taken together, our findings have implications both for theories on prosociality and for society at large. First, results of longitudinal confirmatory factor analyses corroborate our multi-faceted framework of human prosociality that distinguishes altruistically motivated, norm-based, and self-reported human prosociality^34,35^ by demonstrating its validity and stability over time. Together with good re-test reliability in all three sub-components, this finding suggests that our model will be informative to future studies on mediators and moderators of the malleability of prosocial behaviour. Second, differential training effects on sub-components of prosociality further support our motivation-based framework and provide evidence for *plasticity* in the domain of human prosociality. It also challenges neoclassical views in economy advocating fixed and context-insensitive preferences by showing that short daily mental trainings can systematically change social preferences such as altruism in adults^19,20^. Finally, our results identify *which* mental training can effectively shape a multitude of altruistically motivated behaviours, which can inform policy makers and the public alike. Thus, we demonstrate that people’s altruistic motivation and behaviour can be altered through simple, short and non-costly mental practices that target qualities of the heart such as care and compassion. Our findings indicate that stimulating positive prosocial emotions with those in need is a viable alternative to strategies that aim to increase altruism by fostering cognitive understanding and rationality, offer monetary incentives (e.g., tax incentives, as oftentimes proposed by neoclassical views in economy, see^69^), or emphasize the importance of social norms and punishing free riding more rigorously^4^. Cultivating these affective and motivational capacities in schools, in health-related settings, and in work places may be an effective step towards meeting the challenges of a globalized world and move towards global cooperation and a caring society.

## Materials and Methods

### Participants

In total, 332 participants (197 female; mean age = 40.74, SD = 9.24; age range = 20–55) took part in the longitudinal study. Participant recruitment and selection was accomplished by a multi-step procedure including extensive screening for eligibility and adequately informing participants about the requirements of this intense longitudinal training study (see^46^ for detailed description of the recruitment and screening procedure). Participants younger than 20 years or older than 55 years were not admitted to the ReSource Project because children, adolescents, or people of older age would have required different testing protocols (e.g., brain templates, age-adapted tasks, hormone analyses), different equipment (e.g., MRI head coils), and less demanding intervention and testing schedules. The final samples were randomly selected from the pool of eligible participants. First, 191 participants were assigned to Training Cohort 1 (TC1; *N* = 80), Training Cohort 2 (TC2; *N* = 81), or the Retest Control Cohort (RCC1; *N* = 30). Approximately one year later, another 141 participants were recruited and assigned to the Training Cohort 3 (TC3; *N* = 81) or the Retest Control Cohort (RCC2; *N* = 60). Specifically, participants were assigned to cohorts so that cohorts would be matched on demographic variables (age, gender, marital status, income, and IQ) and on self-reported traits (depression, empathy, interoceptive awareness, stress level, compassion for self and others, alexithymia, general mental health, anxiety, agreeableness, conscientiousness, extraversion, neuroticism, and openness) (all ps > 0.1; for details, see^46^). RCC1 and RCC2 were tested separately for logistical reasons, but data (N = 90) were analysed jointly. Including all training and retest control cohorts, 26 participants (7.8%) dropped out during the course of the study (until time point T3; for detailed descriptions of dropout, see Supplement S3 and^46^). Dropout did not differ between training and retest control cohorts.

All participants signed informed consent prior to the study. The study was approved by the Research Ethics Committee of the University of Leipzig, number 376/12-ff and the Research Ethics Committee of the Humboldt University in Berlin, numbers 2013–02, 2013–29, and 2014–10. The study was registered with the Protocol Registration System of ClinicalTrials.gov under the title “Plasticity of the Compassionate Brain” (Identifier: NCT01833104). All experiments were performed and all measures were obtained in accordance with ethical standards of the Declaration of Helsinki.

### Longitudinal design

The prosocial data were part of a large-scale mental training study, the *ReSource Project* (depicted in Fig. 2A; for details see^46^), which aimed to assess differential effects of three distinct 3-months training modules. In particular, the training modules targeted cultivating present-moment focused attention and interoceptive awareness (Presence Module), care, compassion, prosocial motivation, and the ability to deal with difficult emotions (Affect Module), or metacognitive abilities and perspective taking on self and others (Perspective Module). A retest control cohort (RCC) underwent all measurement procedures without any form of mental training. Participants of TC1 and TC2 completed some of the modules in different orders (see Fig. 2A). Specifically, both training cohorts started with the Presence Module, because we consider the training exercises of this module the basis for later socio-affective and socio-cognitive modules. Presence was followed by either the Affect Module (TC1) or the Perspective Module (TC2), before completing the respective other modules. In this way, TC1 and TC2 could serve as active control groups for each other. A third training cohort, TC3, underwent a 3-months Affect Module only, serving as an active control for the Presence Module in TC1 and TC2. All measures of prosociality were assessed at baseline (prior to any training, T0) and during the last 5 weeks of each module (T1-T3, see Fig. 2A). Note that in order to assess the stability of observed effects of mental training, a sub-group of participants was tested again either 4.5 or 10 months after completing the program (T4). In the current paper, we will not report T4 data because the matter of stability will be covered in more detail elsewhere.

### Training modules

Each training module (Presence, Affect, and Perspective) lasted for 13 weeks, starting with a 3-day intensive retreat that was followed by weekly group sessions with teachers as well as daily exercises at home. Exercises were facilitated by a custom-made internet platform and smartphone applications providing audio streams for the guided meditations and an interface for the dyadic exercises. Throughout the retreat, participants were familiarized with the core exercises (see Fig. 2B). During the 2-hour weekly sessions with the teachers, core exercises were discussed and consolidated, and some additional and related practices were taught. The comparably long durations and high intensity of the training modules aimed to ensure that participants could internalize the respective practices and that the specific skills were well consolidated by the time of the testing. No new topics or exercises were introduced during the last 5 weeks of the module (testing phases).

#### Presence Module

The main goal of this module was the cultivation of a deliberate focus of present-moment attention and interoceptive awareness. Core exercises were a Breathing Meditation (focusing attention on one’s breath, refocusing attention when getting distracted^70^) and a Body Scan (systematic engagement and disengagement of attention to sensations in various parts of the body^24^). Additional exercises entailed walking meditation and meditations on primary sensory perception such as vision, sound, and taste.

#### Affect Module

The main aim of this socio-affective module was the cultivation of care and compassion for oneself and others, prosocial motivations, gratitude, and the ability to deal with difficult emotions. Core exercises were a Loving-kindness Meditation^47^ and a newly developed Affect Dyad^71^. During the Loving-kindness Meditation, participants elicited feelings of warmth and care, first towards beloved others, then towards oneself, neutral others, and eventually towards those whom they had difficulties with. The exercise was supported by the mental repetition of phrases like “May you be happy” and “May you live with ease”. The Affect Dyad is a partner exercise that was completed face to face or through an online application. During this contemplative dialogue, participants reported two situations they had recently experienced, one that was experienced as difficult and another that they were grateful for. One participant listened attentively without giving feedback, cultivating empathic listening. The other contemplated the situation without engaging in abstract reasoning or interpretation cultivating familiarization with and acceptance of difficult emotions and developing gratitude. Roles were switched subsequently. Additional exercises were explorations of emotions, Forgiveness Meditation, and development of self-compassion^72^.

#### Perspective Module

The main focus of this socio-cognitive module was the cultivation of metacognitive abilities and perspective taking on oneself and on others. Core exercises were an Observing-thoughts Meditation^73^ and a Perspective Dyad. During the Observing-thought Meditation, participants observed their thoughts in a non-judgmental fashion, labelling them according to the dimensions me/other, past/future, positive/negative, and regarding thoughts as mental phenomena rather than as representations of reality. The Perspective Dyad is a contemplative partner exercise based on the Internal Family System model^74,75^. In the scope of this model, ‘inner parts’ represent stable affective, cognitive, and/or behavioural patterns. For instance, the inner part ‘Manager’ may represent the tendency to structure and organize one’s daily life in a rational manner^74^. Participants identified inner parts throughout the training. During the Perspective Dyad, the speaker described a recently experienced situation from the perspective of a randomly selected inner part (e.g., re-telling a recent job interview from the perspective of one’s inner ‘Clown’). This exercise intends to train the ability to de-couple from an experienced reality and flexibly adopt various different inner perspectives. The listener tried to identify the inner part of the speaker. In order to do so, the listener had to actively engage in cognitively taking the others’ perspective and thus training Theory of Mind capacity. Additional exercises entailed adopting the viewpoint of others who are experienced as very different from oneself and reflections on the central role of thoughts for our behaviour in every-day life.

### Measures of prosociality

We obtained various measures of prosocial behaviour from different research disciplines such as behavioural economics, psychology, and neuroscience. These prosocial measures included game theoretical paradigms, interactive computer tasks, hypothetical distribution tasks, and psychological trait questionnaires^34^. In line with previous work, participants in all behaviour-based measures that entailed real monetary outcomes were informed that they interacted with anonymous others via an online platform. In reality, participants interacted with pre-specified scripts; participants were fully debriefed after the intervention study. For details on each measure, see Table 1.

To keep experimenter demand effects at a minimum, testing procedures were largely computerized and anonymized. In addition, the longitudinal design effectively controls for demand effects by comparing prosocial behaviours in the training cohorts with prosocial behaviours in the retest control cohort, which underwent the exact same testing protocol. Training implementation, data collection, and research were kept strictly separate. Experienced meditation teachers provided the contemplative training interventions, research assistants collected the data, and researchers analysed and interpreted the data.

### Data reduction and measurement invariance testing using longitudinal confirmatory factor analysis

Prosocial measures were integrated according to a previously identified and confirmed 3-factor structure of human prosociality (for illustration, see Fig. 1)^34,35^. We ran multiple time points confirmatory factor analyses (MT-CFA; in SPSS AMOS, version 22) to achieve three goals. First, we aimed to further validate the proposed factor structure by assessing the model fit of all available data of the entire participant sample at four time points (T0-T3). Second, we probed temporal stability of the model and the latent factors of prosociality. Third, we investigated configural, metrical and scalar longitudinal measurement invariance of the model^48,49^, which is a necessary prerequisite for investigating differential and training induced change in the scores of the latent factors of prosociality^48^. To assess longitudinal measurement invariance, we specified our three-factor model^35^ for configural, metric, and scalar invariance testing. In this model, the variables 2^nd^ and 3^rd^ Party Punishment were constrained to equality (see^35^). No residual covariances and cross-loadings were specified (for illustration, see Fig. 1). First, testing whether the structure of prosociality is invariant over time, the *configural invariance model* specified the factor structure and number of items to be the same across time. To account for the fact that measurements were repeatedly obtained from the same participants, the covariances of the indicator errors (autocorrelated errors) were specified across each time point^48^. Additionally, covariances were specified between all latent constructs at each time point and between the different time points for each latent construct (autocorrelated latent constructs). Factor loadings and item intercepts were freely estimated. Subsequently, and based on the configural model, the *metric invariance model* was tested by additionally specifying factor loadings for the same items to be equal over time. Metric invariance tests whether the relation between the items and factors remain stable across time. Finally, and based on the previous models, the *scalar invariance model* was tested by additionally specifying intercepts of the same items to be equal over time. Scalar invariance allows attributing change in the observed measures to change in the underlying construct, rather than to changes in item difficulty or participants’ response criteria^76^. Latent means of the factors after the first time point were estimated freely^48^.

To provide further evidence of the validity of our results, that are based on a relatively small sample size (n = 332), we performed additional analyses of configural, metric, and scalar measurement invariance in models with a reduced number of free parameters. This was achieved by specifying residual covariances (autocorrelation) of the same items to be equal across time^50^.

Model estimation was performed using a full information maximum likelihood (FIML) approach based on unstandardized raw data^77^. Both item- and unit-level missing data over time were low (item-level missing data was 7.0% on average, unit-level missing data was 6.7% on average). Global model fit was assessed using multiple indices^78^: The Tucker-Lewis Index (TLI) and the comparative fit index (CFI) with values greater than 0.90 and 0.95 indicating adequate and good fit respectively^78^ and the root mean square error of approximation (RMSEA) with values lower than 0.05 or lower than 0.08 indicating good or adequate fit^78^. We considered changes in CFI of ≤0.01 when comparing metric invariance models to the configural invariance model and scalar invariance to metric invariance models as indicating given invariance^48^.

### Analysis of training-induced changes using linear mixed-effects models

Analyses were performed with the software Statistical Package for the Social Sciences (SPSS; version 22). The question whether and how the sub-components of prosociality are affected by the different training modules was addressed by means of linear mixed-effects models (LMM) which are robust to unbalanced designs and are able to handle incomplete subject data^51^. Specifically, these models use a full information maximum likelihood approach to missing data, which allows the most accurate estimation of effects and unbiased results. In addition, variables related to dropout can be included as covariates in LMMs in order to control for selective attrition. Please note that dropout was rather low in our study (7.8%) and did not differ between training and retest cohorts (see also Supplementary Table S3). Also, none of the demographic and self-reported variables assessed at T0 were systematically related to dropout in our study (for details, see^46^). Please note that the calculation of Lee bounds^79^ to further control for selective attrition was precluded in our study by the violation of the monotonicity assumption (i.e., the assumption that assignment to treatment versus retest affects attrition only in one direction).

Based on results of the longitudinal CFA, scores for each factor of prosociality were created for each time point and each participant by integrating the individual prosocial measures that belong to the respective factors. Specifically, all individual prosocial measures were z-transformed across all testing phases (T0 to T3; preserving relative differences between time points) and then the transformed measures of prosociality were averaged according to the three factors for each time point, so that each participant received one score for each factor of prosociality and each time point. To investigate whether these prosocial factor scores develop differentially over time and between cohorts, we specified a LMM for each sub-component for the between-subject factor *group* (4 levels: RCC, TC1, TC2, TC3), the within-subject factor *time* (4 levels: T0, T1, T2, T3), and random intercepts for participants. Continuous time was added as a repeated statement with the AR(1) covariance structure. Gender and age were included as control variables.

Within this model, we first tested whether baseline levels of the factors of prosociality were comparable across cohorts, that is, whether there was no significant effect of *group* at T0. Second, within the same model, we assessed whether there was a significant two-way interaction of *group* and *time*, which would suggest differential changes over time between the different cohorts. In case of significant two-way interactions, we further examined if effects of *time* were found in retest control or training cohorts. In case of significant changes in factor scores over time for training cohorts, we also investigated which training module (Presence, Affect, Perspective) was driving this effect. Specifically, overall effects of a training module were calculated as the average increase in factor scores due to this particular module. For instance, in TC1, difference scores of pre- and post-module factor scores were estimated as follows: Presence Module: T1 minus T0; Affect Module: T2 minus T1; Perspective Module: T3 minus T2 (see Fig. 2A). Module-specific effects were similarly calculated for TC2 and TC3 and averaged across testing cohorts. Hence, for each factor of prosociality, the overall effect of the Presence Module was calculated as the average of T1 minus T0 in TC1 and TC2. The overall effect of the Affect Module was calculated as the average of T2 minus T1 in TC1, T3 minus T2 in TC2, and T1 minus T0 in TC3. The overall effect of the Perspective Module was calculated as average of T3 minus T2 in TC1, and T2 minus T1 in TC2. Based on these measures of module-specific change in prosocial behaviour, we investigated if a particular module significantly affected factor scores of prosociality. For each prosocial factor, module-specific effects were tested against zero (representing the null-hypothesis of no training-induced change in prosocial factor scores; one sample t-test, two-tailed) and against the average change in factor scores in the RCC from T0 to T3 (independent sample t-test, two-tailed). Benjamini-Hochberg corrections^52^ were applied to correct for multiple t-tests. Cohen’s d provides a measure of effect size.

In case of significant training effects for particular modules, we also tested whether the order of the training modules mattered, that is, whether the respective modules had differential effects depending on the order of the trainings.

## Electronic supplementary material

Supplementary Material

## Data Availability

The datasets generated and analysed during the current study are available at request for replication purposes and after signing a data sharing agreement.
