# Supplementary material for: Distinct mental trainings differentially affect altruistically motivated, norm motivated, and self-reported prosocial behaviour
Source: Sci Rep. 2018 Sep 10;8:13560. doi: 10.1038/s41598-018-31813-8 (PMC6131389; doi:10.1038/s41598-018-31813-8)
Supplement: Supplementary file 1 — Supplementary Material [file 41598_2018_31813_MOESM1_ESM.pdf]

Supplementary Materials

Distinct mental trainings differentially affect altruistically motivated, norm motivated, and self-reported prosocial behaviour

Anne Böckler, Anita Tusche, Peter Schmidt, Tania Singer

Supplementary Table S1: Descriptive statistics for all measures of prosociality obtained at T0-T3, depicted separately for each experimental cohort.

Supplementary Table S2a: Standardized factor loadings for individual measures of prosociality in the scalar invariance model at all time points.

Supplementary Table S2b: Unstandardized factor loadings for individual measures of prosociality in the scalar invariance model at all time points.

Supplementary Table S2c: Coefficients of autocorrelations for latent factors across time in the scalar invariance model.

Supplementary Table S2d: Coefficients for latent factors correlations within each time point.

Supplementary Table S3: Details on drop out and missing data for all measurement time points.

Supplement S4: Results for individual measures of strategic prosocial behavior.

Supplemet S5: Results for module comparisons

Supplementary Table S1: Means and standard deviations (SD) for all measures of prosociality obtained at T0 to T3, depicted separately for each experimental cohort.

		T0			T1			T2			T3	
	N	Mean	SD	N	Mean	SD	N	Mean	SD	N	Mean	SD
**Group 1 - TC1**												
DG	81	34.86	17.18	77	33.73	17.59	76	35.21	17.55	73	37.53	18.22
Strategic giving	81	4.91	18.69	77	8.16	19.66	76	6.02	16.75	73	3.01	15.99
TG	81	39.16	30.24	77	36.11	26.39	76	35.71	29.38	73	35.81	28.57
2nd PPG	81	27.97	22.05	77	24.12	22.17	76	19.38	18.70	73	19.41	19.45
3rd PPG	81	30.11	22.71	77	30.08	23.36	76	24.85	23.31	73	22.02	22.21
ZPG helping	78	61.15	27.21	77	68.83	22.88	75	66.93	23.07	73	63.97	23.91
ZPG cost effect	78	3.21	31.56	77	16.88	32.30	75	20.33	35.76	73	24.66	35.96
Social Discounting	68	0.05	0.10	64	0.07	0.11	68	0.09	0.19	64	0.05	0.12
SVO	79	6.28	3.56	73	6.33	3.64	76	6.89	3.42	74	6.85	3.58
Donation	79	27.88	14.26	79	30.12	14.29	77	33.39	14.37	73	31.90	15.04
Prosocial Scale	79	3.27	0.49	73	3.45	0.54	77	3.46	0.56	74	3.55	0.52
Machiavelli Index	79	2.96	2.34	73	2.62	2.53	76	2.00	1.91	74	1.91	1.86
IRI	80	22.28	2.48	73	22.49	2.57	77	22.69	2.42	74	22.77	2.33
**Group 2 - TC2**												
DG	78	32.83	18.09	77	33.00	16.90	73	33.46	19.16	74	35.94	16.23
Strategic giving	78	11.74	23.33	77	10.93	18.52	73	7.96	20.36	74	8.03	15.05
TG	78	40.55	29.91	77	42.05	32.12	73	41.82	32.80	74	43.16	33.15
2nd PPG	78	28.77	22.24	77	24.96	21.83	73	24.29	21.99	74	21.67	20.56
3rd PPG	78	33.70	24.52	77	31.56	25.00	73	29.00	24.63	74	27.93	24.94
ZPG helping	78	63.59	26.18	72	68.61	23.93	73	68.77	21.60	74	67.43	22.82
ZPG cost_effect	78	14.74	28.90	72	16.32	32.81	73	17.81	38.30	74	23.99	36.41
Social Discounting	61	0.05	0.05	73	0.08	0.14	72	0.05	0.07	69	0.06	0.12
SVO	79	5.92	3.54	75	6.59	3.33	75	6.44	3.55	57	7.33	3.03
Donation	78	26.99	14.70	77	30.11	13.41	75	32.18	14.90	71	35.09	14.26
Prosocial Scale	79	3.34	0.54	76	3.47	0.53	73	3.64	0.50	74	3.73	0.53
Machiavelli Index	79	2.80	2.23	77	2.84	2.44	73	2.70	2.60	74	1.95	2.34
IRI	80	22.87	2.21	76	22.55	2.39	73	23.11	2.32	74	23.18	2.21

**Group 3 - TC3**												
DG	80	35.53	15.29	77	36.06	17.08						
Strategic giving	80	5.91	15.59	77	6.98	19.22						
TG	80	43.02	31.21	77	42.43	31.63						
2nd PPG	80	30.41	20.64	77	24.42	21.50						
3rd PPG	80	30.57	22.11	77	27.13	24.22						
ZPG helping	81	69.88	22.89	76	69.74	25.35						
ZPG cost_effect	81	6.79	31.38	76	10.53	33.73						
Social Discounting	70	0.05	0.08	69	0.04	0.05						
SVO	79	6.05	3.65	76	6.91	3.01						
Donation	80	29.92	14.92	78	34.54	12.92						
Prosocial Scale	79	3.52	0.59	76	3.49	0.53						
Machiavelli Index	79	2.46	2.49	76	1.97	1.87						
IRI	79	22.29	2.38	76	22.69	2.15						
**Group 0/4 - RCC**												
DG	88	33.32	18.63	83	30.49	18.49	82	29.08	18.87	79	29.19	18.70
Strategic giving	88	7.21	25.29	83	10.97	18.00	82	5.85	19.10	79	6.31	23.08
TG	88	41.88	31.90	83	37.43	30.66	82	29.59	22.40	79	33.57	30.69
2nd PP	88	32.79	23.63	83	31.20	21.57	82	27.18	21.07	79	24.71	20.62
3rd PP	88	35.26	21.88	82	34.34	22.59	82	29.59	22.40	79	28.56	22.95
ZPG helping	89	64.16	23.15	84	63.69	24.39	80	66.13	20.28	78	65.00	20.37
ZPG cost_effect	89	9.27	30.94	84	13.10	34.38	80	31.88	36.88	78	35.26	41.18
Social Discounting	77	0.10	0.15	74	0.10	0.13	70	0.11	0.18	66	0.11	0.16
SVO	85	5.53	3.91	84	5.88	3.77	79	5.90	3.77	79	6.66	3.52
Donation	88	26.42	12.49	83	27.28	12.96	82	27.10	14.87	78	26.89	14.99
Prosocial Scale	85	3.39	0.59	84	3.43	0.55	79	3.46	0.61	79	3.47	0.58
Machiavelli Index	85	3.47	3.20	84	3.51	3.18	79	3.43	3.45	79	3.24	3.70
IRI	86	22.65	2.35	84	22.25	2.57	78	22.13	2.50	79	22.23	2.34

DG =Dictator Game, TG = Trust Game, 2nd PPG = 2nd Person Punishment Game, 3rd PPG = 3rd Person Punishment Game, ZPG = Zurich Prosocial Game, SVO = Social Value Orientation, IRI = Interpersonal Reactivity Index

Supplements S2: Model specification and solution for multiple time points confirmatory factor analyses (MT-CFAs)

Supplementary Table S2a: Standardized factor loadings (β) for individual measures of prosociality in the scalar invariance model at all time points. All factor loadings were significant. Note that the relation between the measure Social Discounting and the factor Self-Reported Prosocial Behaviour was not modelled in present analyses, as it was only added in a data-driven manner in (*2*, *3*) .

	T0	T1	T2	T3
Altruistically Motivated Prosocial Behaviour				
ZPG Helping	.372	.388	.472	.482
SVO	.467	.513	.539	.571
Social Discounting	-.417	-.412	-.347	-.401
TG	.421	.442	.495	.498
Donation	.373	.414	.427	.428
DG	.610	.608	.651	.710
Norm Motivated Prosocial Behaviour				
2nd Party Punishment	.779	.862	.901	.894
3rd Party Punishment	.784	.801	.785	.776
Self-Reported Prosocial Behaviour				
Prosocialness Scal	.868	.839	.799	.891
IRI	.420	.379	.383	.440
Machiavelli Scale	-.317	-.308	-.293	-.317

DG =Dictator Game, TG = Trust Game, 2nd PPG = 2nd Person Punishment Game, 3rd PPG = 3rd Person Punishment Game, ZPG = Zurich Prosocial Game, SVO = Social Value Orientation, IRI = Interpersonal Reactivity Index

Supplementary Table S2b: Unstandardized factor loadings (β) for individual measures of prosociality in the scalar invariance model at all time points. All factor loadings were significant.

	T0	T1	T2	T3
Altruistically Motivated Prosocial Behaviour				
ZPG Helping	.858	.858	.858	.858
SVO	.160	.160	.160	.160
Social Discounting	-.004	-.004	-.004	-.004
TG	1.222	1.222	1.222	1.222
Donation	.511	.511	.511	.511
DG	1.000	1.000	1.000	1.000
Norm Motivated Prosocial Behaviour				
2nd Party Punishment	1.000	1.000	1.000	1.000
3rd Party Punishment	1.000	1.000	1.000	1.000
Self-Reported Prosocial Behaviour				
Prosocialness Scal	.497	.497	.497	.497
IRI	1.000	1.000	1.000	1.000
Machiavelli Scale	-.865	-.865	-.865	-.865

Supplementary Table S2c: Coefficients of autocorrelations for latent factors across time in the scalar invariance model. All correlations were significant.

	T0	T1	T2
Altruistically Motivated Prosocial Behaviour			
T1	.896		
T2	.827	.877	
T3	.771	.803	.952
Norm Motivated Prosocial Behaviour			
T1	.686		
T2	.617	.777	
T3	.531	.773	.850
Self-Reported Prosocial Behaviour			
T1	.923		
T2	.897	.979	
T3	.696	.805	.883

Supplementary Table S2d: Coefficients for latent factors correlations within each time point. No correlations were significant.

	T0	T1	T2	T3
AMPB – NMPB	.065	.060	.050	-.021
AMPB – SRPB	.067	.023	-.030	.002
NMPB – SRPB	.036	.034	-.034	-.001

AMPB = Altruistically Motivated Prosocial Behaviour; NMPB = Norm Motivated Prosocial Behaviour; SRPB = Self-Reported Prosocial Behaviour

Supplementary Table S3: Reasons for missing data within the *ReSource Project* in the context of which the present study was conducted.

Reasons	*N*
Participants originally enrolled in the study	332
Drop out during T0	2
Drop out during T1	10
Drop out during T2	7
Drop out during T3	7
Participants with missing measurement(s) at T0 due to scheduling/technical problems	16
Participants with missing measurement(s) at T1 due to scheduling/technical problems	22
Participants with missing measurement(s) at T2 due to scheduling/technical problems	14
Participants with missing measurement(s) at T3 due to scheduling/technical problems	9
Missing data in the social discounting task due to 'multiple cross-over'* or outlier at T0	56
Missing data in the social discounting task due to 'multiple cross-over' or outlier at T1	36
Missing data in the social discounting task due to 'multiple cross-over' or outlier at T2	19
Missing data in the social discounting task due to 'multiple cross-over' or outlier at T3	24

* see Jones & Rachlin, 2006

Study dropout did not differ between TC1, TC2, TC3 and RCC: T0 (TC1, TC2, TC3, RCC): 0, 0, 0, 2; T1: 2, 2, 3, 3; T2: 2 3, -, 2; T3: 4, 1, -, 2.

Supplement S4: Results for individual measures of strategic prosocial behaviour.

The original model of human prosociality also included measures assessing *strategic* prosocial behaviour, related to the inclination to make prosocial choices dependent upon whether they benefit oneself (*2*). However, further investigation is required to confirm whether a separate strategy factor can be established (*3*). For the sake of completeness, we provide details on individual measures of strategic prosocial behaviour included in the ReSource Project and their training-induced plasticity.

Training-induced changes in prosociality

Cost-effect. No significant differences in the cost-effect were revealed at baseline (F(3, 822.75) = 1.76, p = .154). The LMM revealed a main effect of *time* (F(3, 580.32) = 17.98, p = .000), reflecting increases in cost-benefit oriented behaviour over the course of the study. The main effect of *group* was not significant (F < 1.04). However, the two-way interaction of *time* and *group* reached significance (F(7, 594.40) = 2.67, p = .010), due to a strong increase in the cost-effect over time in the RCC (F(3, 489.37) = 14.95, p = .000), but no such effect in the majority of training cohorts (TC1: F(3, 492.57) = 8.19, p = .000; TC2: F(3, 497.64) = 1.37, p = .250; TC3: F(1, 659.87) = 0.66, p = .417).

Module-specific analyses showed that the increase in the cost-effect over time was significantly different from zero with a medium effect-size in the RCC (t(83) = 5.14, p = 000, d = .56), suggesting increasing strategic behaviour in this group. A similar, though weaker, effect was found after the Presence Module (t(144) = 2.37, p = .019, d = .20; surviving correction for multiple comparisons), but not after the other training modules (ts ≤ 1.53, ps ≥ .126). When comparing the increase in cost-benefit oriented behaviour in the RCC and training modules, no significant differences were found, however (ts ≤ 1.70, ps ≥ .090). These results indicate that over the course

of the study, participants increasingly incorporated cost-benefit calculations into their decisions, a tendency that was especially prevalent in the RCC. The absence of a respective increase in most of the training cohorts and after the later modules (Affect and Perspective) suggest that continuous mental training may buffer the increase of strategic behaviour and seld-interest that is otherwise observed when people become familiar with the tasks (and how to increase one's personal gains).

Strategic giving. No significant differences in strategic giving were revealed at baseline ($F(3, 1037.19) = 1.97$, $p = .117$). The LMM revealed a marginal effect of *time* ($F(3, 654.50) = 2.51$, $p = .058$), reflecting a tendency for decreases in strategic giving over the course of the study. The main effect of *group* was not significant ($F(3, 317.20) = 2.25$, $p = .082$) and neither the two-way interaction of *time* and *group* ($F < 1$). These findings suggest that strategic giving is not altered due to any of the mental trainings.

Correlations between training-induced changes in prosociality

We found a significant correlation between training-induced increases in altruistically motivated prosocial behaviour and training-induced reductions of the cost-effect in the ZPG ($r = -.15$, $p = .022$; surviving correction for multiple comparisons). This finding suggests that the more the meditation based training induces altruistic motivations, the more it reduces self-centred strategic considerations when faced with other's needs. Hence, people who became more motivated to enhance others' well-being over the course of the training became also less concerned with their own (monetary) advantage in strategic cooperative settings. This finding may point towards a more general antagonistic relationship between altruism and egoism, in that shifts *towards* the motivation to care for and support others (*4*, *5*) may be inherently linked to shifts *away* from the motivation to maximize self-interest and own ressources (*5*, *6*).

Supplement S5: Results for module comparisons.

The main aim of the present study was to investigate which mental training modules effectively enhance which facet of prosociality and, consequently, we compared effects of the three mental training modules to changes in the RCC. To directly address differences between the three training modules (independent of RCC), we performed additional LMMs for each sub-component of prosociality, specifying the factors *module* (4 levels: Baseline, Presence, Affect, Perspective), *time* (4 levels: T0, T1, T2, T3), and random intercepts for participants. Continuous time was added as a repeated statement with the AR(1) covariance structure. Gender and age were included as control variables.

Altruistically Motivated Prosocial Behaviour. Results of the LMM showed no significant main effect of *module* and no two-way interaction of *time* and *module* (Fs < 1.58, ps ≥ .20). This finding suggests that though only the Affect Module significantly enhanced altruistic behaviours above changes in the RCC, training effects did not differ significantly between the modules.

Norm Motivated Prosocial Behaviour. Results of the LMM revealed neither a significant main effect of *module* nor a two-way interaction of *time* and *module* (Fs < 2.62, ps ≥ .10). This finding further supports previous results, indicating that norm-based behaviours were not (differentially) altered by any form of mental training.

Self-Reported Prosocial Behaviour. Neither the main effect of *module* nor the two-way interaction of *time* and *module* reached significance (Fs < 1.1, ps ≥ .36). Hence, the training-induced increases in self-reported prosociality did not differ between the training modules.

Strategic Behaviour (i.e. Cost-effect and strategic giving). For the Cost-effect, results of the LMM showed no significant main effect of *module* and no two-way interaction of *time* and *module* (Fs < 1.05, ps ≥ .31). Similarly, there was neither a significant main effect of *module* nor a two-

way interaction of *time* and *module* for strategic giving ($Fs < 1.45$, $ps \geq .23$). These results suggest that the training modules did not differ regarding their influence on strategic behaviours.

References

1. B. Jones, H. Rachlin, Social discounting. *Psychological science* **17**, 283-286 (2006).
2. A. Böckler, A. Tusche, T. Singer, The Structure of Human Prosociality Differentiating Altruistically Motivated, Norm Motivated, Strategically Motivated, and Self-Reported Prosocial Behavior. *Social Psychological and Personality Science*, 1948550616639650 (2016).
3. A. Böckler, A. Tusche, T. Singer, The Structure of Human Prosociality Revisited: Corrigendum and Addendum to Böckler, Tusche, and Singer (2016). *Social Psychological and Personality Science*, 1948550617722200 (2017).
4. W. D. Hamilton, The general evolution of social behavior. II. *Journal of Theoretical Biology* **7**, 17-52 (1964).
5. J. Heckhausen, Evolutionary perspectives on human motivation. *Am Behav Sci* **43**, 1015-1029 (2000).
6. R. Dawkins, *The Selfish gene*. (Oxford University Press, New York, N.Y., 1976).
